# Crystalline-Amorphous-Crystalline Transformation in a Highly Brilliant Luminescent System with Trigonal-Planar Gold(I) Centers

**DOI:** 10.1038/srep26002

**Published:** 2016-05-17

**Authors:** Kosuke Igawa, Nobuto Yoshinari, Mitsutaka Okumura, Hiroyoshi Ohtsu, Masaki Kawano, Takumi Konno

**Affiliations:** 1 Department of Chemistry, Graduate School of Science, Osaka University, Toyonaka, Osaka 560-0043, Japan; 2 The Division of Advanced Materials Science, Pohang University of Science and Technology (POSTECH), San 31, Hyoja-dong, Pohang 790-784, Korea; 3 Department of Chemistry, Graduate School of Science and Engineering, Tokyo Institute of Technology, 2-12-1 Ookayama, Meguro-ku, Tokyo 152-8550, Japan

## Abstract

Photoluminescent compounds showing emission color changes in response to external stimuli have received considerable attention because of their wide range of applications. Here, we report the unique photoluminescence behavior of a digold(I) coordination system with trigonal-planar Au^I^ centers, [Au_2_(dppm)_3_]^2+^ (dppm = bis(diphenylphosphino)methane). This system shows an extremely intense phosphorescence, with a quantum yield of >95% in the solid state. Both the emission color and thermal stability vary due to changing counter ions (Cl^−^ vs. OTf^−^). Of particular note is the thermal crystalline-amorphous-crystalline transformation for the chloride salt, which is accompanied by drastic emission color changes. Single-crystal and powder X-ray diffractions demonstrate that the two-step transformation is induced by the loss of water molecules of crystallization with the subsequent removal of a dppm ligand to form [Au_2_(dppm)_2_]^2+^, which is mechanically reverted back to [Au_2_(dppm)_3_]^2+^.

Chromic luminescent compounds whose emission energies and intensities change in response to external stimuli have received increasing attention because of their potential applications as chemical/biological probes and organic light-emitting diodes^1^,^2^,^3^. These compounds often involve Au^I^ ions as a metal component^4^,^5^,^6^,^7^,^8^,^9^, in which their photo-luminescence is highly sensitive to the change in intramolecular and intermolecular Au···Au aurophilic interactions^10^,^11^. Thus far, organic vapors^4^, solvents^5^, counter anions^6^, metal ions^7^, temperature^8^, and mechanical force^9^ have been used as triggers to change the emission colors and intensities of gold(I) compounds. Generally, each Au^I^ center of these compounds has a two-coordinated linear geometry^12^,^13^. This geometry enables them to form an effective Au···Au interaction and to accept another donor that exists as a counter ion or solvent molecule to their vacant coordination sites. In contrast, the chromic behavior has not been reported for those containing three-coordinated, trigonal-planar Au^I^ centers^14^,^15^,^16^. This is surprising because three-coordinated gold(I) species are expected to show a stronger photo-luminescence due to the steric crowdedness around each Au^I^ center, which can inhibit non-radiative processes^17^. In this context, we started to investigate the photo-luminescent properties of a typical digold(I) complex, [Au_2_(dppm)_3_]^2+^ (dppm = bis(diphenylphosphino)metane, [1]^2+^), in which two Au^I^ centers are triply bridged by three dppm ligands. This complex was prepared and isolated as bromide and perchlorate salts by McAuliffe and coworkers in 1985, who proposed that each Au^I^ center in [1]^2+^ has a trigonal-planar geometry based on the ^31^P NMR spectroscopic and elemental analytical results^18^. However, its crystal structure and emission property have not yet been investigated. In this study, we succeeded in the crystallization and structural characterization of the chloride and trifluoromethanesulfonate (OTf^−^) salts of [1]^2+^. In the solid state, both salts exhibit a quite brilliant emission, green for the chloride salt and yellow-green for the OTf^−^ salt, with a quantum yield of >95%. More remarkably, a reversible, two-step emission color change from green to blue via yellow, induced by the loss of water molecules of crystallization with the subsequent loss of one of three bridging dppm ligands to form [Au_2_(dppm)_2_]^2+^ ([2]^2+^), was detected for the chloride salt in the solid state (Fig. 1). To our knowledge, such a fascinating chromic luminescence has never been reported for gold(I) compounds with trigonal-planer Au^I^ centers.

## Results

### Synthesis and structural characterization of [1]Cl_2_∙8.5H_2_O and [1](OTf)_2_∙H_2_O

Treatment of [Au_2_Cl_2_(dppm)]^19^ with dppm at a 1:2 ratio in MeOH/H_2_O produced a clear yellow solution, from which the chloride salt of [1]^2+^ ([1]Cl_2_·8.5H_2_O) was isolated as pale yellow block crystals at a high yield. The OTf^−^ salt of [1]^2+^ ([1](OTf)_2_·H_2_O) was also obtained as pale yellow platelet crystals by adding NaOTf to the yellow reaction solution. The elemental and thermogravimetric (TG) analytical data implied that freshly prepared crystals of the chloride and OTf^−^ salts contained 8.5 water molecules and one water molecule per one complex cation, respectively. The presence of water molecules in each compound was confirmed by IR spectroscopy, showing a broad band at approximately 3400 cm^−1^ (Supplementary Fig. 1)^20^.

**Figure 2:**
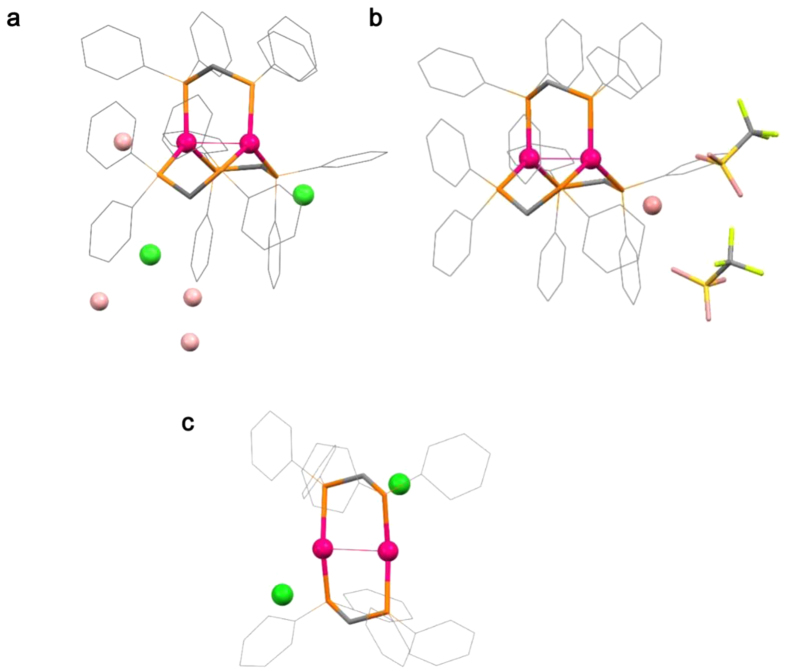
Crystal structures of [1]^2+^ and [2]^2+^. Perspective views of (**a**), [1]Cl_2_·8.5H_2_O, (**b**) [1](OTf)_2_·H_2_O and (**c**) [2]Cl_2_. Color Codes: Au, red; P, orange; S, yellow; Cl, green; C, gray; O, pink; F, light green.

Single-crystal X-ray crystallography indicated that [1]Cl_2_·8.5H_2_O is crystallized in a cubic space group *Pa*-3, consisting of one third of the complex cation of [1]^2+^, two thirds of chloride anions, and two and five sixths of water molecules of crystallization in the asymmetric unit. The entire complex cation contains two Au^I^ ions that are triply bridged by three dppm ligands, forming a digold(I) structure in [Au_2_(dppm)_3_]^2+^ (Fig. 2a). Each Au^I^ ion, which lies on a crystallographic *C*_3_ axis, is coordinated by three P atoms from three different dppm ligands in an ideal trigonal-planar geometry (av. Au–P = 2.41 Å and P–Au–P = 120°). This triply bridged structure in [1]^2+^ is reminiscent of that found in the corresponding disilver(I) complex, [Ag_2_(dppm)_3_]^2+^ 21. The intramolecular separation between two Au^I^ centers in [1]Cl_2_·8.5H_2_O is 3.03 Å, suggestive of the presence of an aurophilic interaction. In crystal, there exist intermolecular CH···π interactions between complex cations (C···Ph = 3.07–3.50 Å). Moreover, two Cl^−^ counter anions and water molecules of crystallization are located around each complex cation, forming CH···Cl (C···Cl = 3.56–3.81 Å) and OH···Cl (O···Cl = 2.91–2.96 Å) interactions.

Single-crystal X-ray analysis indicated that [1](OTf)_2_·H_2_O is crystallized in a monoclinic space group *P*2_1_/*n*, consisting of two complex cations of [1]^2+^ (av. Au–P = 2.39 Å and av. P–Au–P = 120°), four OTf^−^ anions, and two water molecules of crystallization in the asymmetric unit. The digold(I) structure of each complex cation in this compound is nearly the same as that found in [1]Cl_2_·8.5H_2_O (Fig. 2b). The intramolecular separations between two Au^I^ centers in the two complex cations are 2.97 and 2.99 Å, which are slightly shorter than the separation in [1]Cl_2_·8.5H_2_O (3.03 Å). The relatively strong CH···O/F (C···O = 3.06–3.30 Å, C···F = 3.18–3.58 Å) interactions between a complex cation and OTf^−^ anion, as well as intermolecular CH···π interactions between complex cations (C···Ph = 3.17–3.61 Å), are found in [1](OTf)_2_·H_2_O.

### Photoluminescence behavior of [1]Cl_2_·8.5H_2_O and [1](OTf)_2_·H_2_O

**Figure 3:**
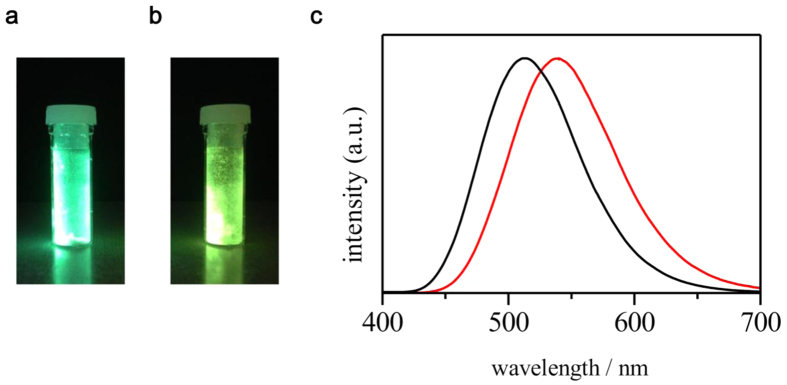
Phosphorescence of [1]Cl_2_ and [1](OTf)_2_. Photographs of (**a**), [1]Cl_2_·8.5H_2_O, and (**b**) [1](OTf)_2_·H_2_O under UV light. (**c**) Emission spectra of [1]Cl_2_ (black) and [1](OTf)_2_ (red) in the solid state at room temperature. (λ_ex_ = 390 nm)

Under UV light irradiation at room temperature, solid samples of [1]Cl_2_·8.5H_2_O and [1](OTf)_2_·H_2_O showed strong green and yellow-green emissions, respectively (Fig. 3a,b). In the emission spectra, [1]Cl_2_·8.5H_2_O and [1](OTf)_2_·H_2_O showed broad bands centered at 413 and 440 nm, respectively. The emission lifetimes for [1]Cl_2_·8.5H_2_O and [1](OTf)_2_·H_2_O were estimated to be 4.51 μs and 4.15 μs, respectively (Supplementary Fig. 2), indicative of the phosphorescent character of emission for both compounds. Time-dependent density functional theory (TD-DFT) calculations of [1]Cl_2_·H_2_O showed intense absorption at 325.6 and 324.8 nm, which involved one-electron transitions from HOMO-11 to LUMO and HOMO-12 to LUMO, respectively. These MOs possessed a large contribution from the Au···Au core (Supplementary Figs 3 and 4). Moreover, the DFT calculations confirmed that the luminescence of this compound has a phosphorescent character due to an intersystem crossing from the lowest singlet excited (S_1_) state to the lowest triplet excited (T) state; the calculated emission wavelength (511 nm) of the luminescence was highly similar to the experimental one. Notably, the internal luminescence quantum yields (Φ) for both salts were estimated to be more than 95% based on the absolute method using an integrating sphere. This value is considerably greater than that for the corresponding doubly bridged digold(I) complex, [Au_2_Cl_2_(dppm)_2_]^12^; the quantum yield of this complex was estimated to be 69% (λ_em_ = 480 nm) under the same conditions. The emission band of [1]Cl_2_·8.5H_2_O and [1](OTf)_2_·H_2_O shifted slightly (by 10–15 nm) to longer wavelength when the temperature was lowered to 77 K, indicative of a stronger Au···Au interaction in the excitation state at lower temperature (Supplementary Fig. 5). In addition, the emission lifetime (ca. 5 μs) observed at 77 K was nearly the same as that (ca. 4 μs) observed at room temperature (Supplementary Fig. 2), and the quantum yields of [1]Cl_2_·8.5H_2_O and [1](OTf)_2_·H_2_O were still very high at 77 K. [1]Cl_2_·8.5H_2_O and [1](OTf)_2_·H_2_O dissolved in methanol showed a weak yellow emission (λ_em_ = 592 nm, Φ < 1%, Supplementary Fig. 6), presumably because of the equilibrium between the triply bridged structure in [1]^2+^ and the doubly bridged structure in [Au_2_(dppm)_2_]^2+^ ([2]^2+^) in the solution^22^.

### Two-step thermal transformation with emission color change

**Figure 4:**
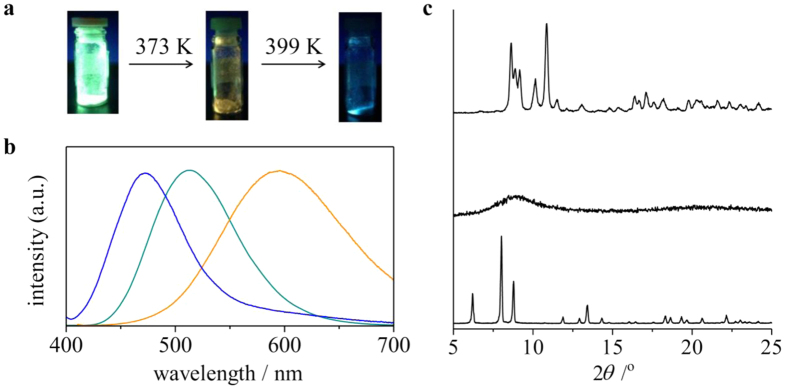
Emission color changes of [1]Cl_2_·8.5H_2_O by heating. (**a**) Photographs under UV light and (**b**) emission spectra of fresh (green line) and heated samples (treated at 373 K, yellow line; at 399 K, blue line) of [1]Cl_2_·8.5H_2_O.

The TG analysis of [1]Cl_2_·8.5H_2_O indicated that a gradual weight loss of 8.0%, which corresponds to the loss of 8.5 water molecules, occurred until 373 K to give a dehydrated form, followed by its decomposition at 573 K (Supplementary Fig. 7). Although no weight loss was observed between 373 and 473 K, the differential scanning calorimetry (DSC) showed an exothermal peak at 399 K, indicative of the occurrence of an endothermal reaction at this temperature. In parallel with the dehydration reaction and subsequent endothermal reactions, dramatic changes in the original emission color were recognized; the emission color changed from green to yellow when a solid sample of [1]Cl_2_·8.5H_2_O was heated at 373 K, and further heating to 399 K caused the color change to blue (Fig. 4a). In the emission spectra, the sample heated at 373 K showed an emission band at 596 nm with a quantum yield of 52%, whereas the sample heated at 399 K showed an emission band at 470 nm with a quantum yield of 55% (Fig. 4b).

We carried out a solid-state ^31^P NMR spectroscopy and powder X-ray diffraction (PXRD) study to characterize the two species formed by heating at 373 and 399 K. The ^31^P NMR spectrum of an original solid sample of [1]Cl_2_·8.5H_2_O showed a signal centered at δ 18 ppm (Supplementary Fig. 8), which was assigned to P donors bound to Au^I^ centers. The same NMR spectral feature was observed for the sample heated at 373 K, indicative of the retention of the digold(I) structure in [1]^2+^ by dehydration. In contrast, the sample heated at 399 K exhibited a new signal at δ −44 ppm. Because the signal at δ −44 ppm corresponds well with that observed for a solid sample of dppm, the heating of [1]Cl_2_ at 399 K causes the dissociation of a part of dppm ligands in [1]^2+^. In the PXRD, the diffraction pattern of a solid sample of [1]Cl_2_·8.5H_2_O was consistent with that simulated from its single-crystal X-ray data. In contrast, no notable diffractions were observed for a sample heated at 373 K. This is indicative of the collapse of the non-covalent intermolecular interactions due to the removal of water molecules of crystallization, thus converting them to an amorphous solid. Remarkably, the PXRD for a sample heated until 399 K exhibited sharp diffractions that were distinct from those for [1]Cl_2_·8.5H_2_O. The structure of this new crystalline phase was successfully determined by the high-resolution powder X-ray diffraction using synchrotron radiation (λ = 1.30 Å) (Fig. 2c, Supplementary Fig. 9), which revealed the presence of a complex cation of [2]^2+^ and two chloride ions in the asymmetric unit. This complex cation contains two linear Au^I^ ions that are bridged by two dppm ligands, and each Au^I^ center has a diagonal P–Au^I^–P geometry (av. Au–P = 2.34 Å and P–Au–P = 159°), with an Au···Au separation of 2.97 Å. In this structure, Cl^−^ ions are not involved in the coordination (Au···Cl > 3.6 Å) but act as a counter anion with the formation of CH···Cl interactions with neighboring complex cations. This structure is in sharp contrast to the previously reported structure in [Au_2_(dppm)_2_Cl_2_]∙acetone^23^, in which each Cl^−^ ion weakly coordinates to an Au^I^ center (av. Au–Cl = 2.77 Å) to form a *T*-shaped coordination geometry (Supplementary Fig. 10). Moreover, complex cations are connected to each other through multiple CH···π or π···π interactions, forming a closely packed lattice structure. Whereas non-coordinating dppm molecules are not accommodated in this crystal lattice, the elemental analytical data and the solid-state ^31^P NMR spectrum implied that the dppm molecules were not sublimated to air but were still contaminated as an amorphous solid in the sample heated at 399 K.

When a blue-emissive crystalline powder of [2]Cl_2_ was manually ground in an agate mortar, a yellow-emissive amorphous powder of [1]Cl_2_ was produced. Moreover, the continuous grinding of the powder of [1]Cl_2_ after adding water produced a green-emissive crystalline powder of [1]Cl_2_·8.5H_2_O. The assignment of these powders was made using their emission spectra and PXRD profiles (Supplementary Figs 11 and 12), thus indicating the reverse conversion from [2]Cl_2_ to [1]Cl_2_ and then to [1]Cl_2_·8.5H_2_O. Here, no significant change in the emission color was observed for [1](OTf)_2_·H_2_O, even when its sample was heated to 473 K. Furthermore, the emission spectrum and PXRD pattern of a sample heated at 473 K were essentially identical to those of the original sample (Supplementary Figs 13 and 14), illustrating that its thermal stability is considerably higher than that of the OTf^−^ salt of [1]^2+^.

## Discussion

We succeeded in the crystallization and structural characterization of [1]Cl_2_·8.5H_2_O and [1](OTf)_2_·H_2_O, in which two trigonal-planar Au^I^ centers are triply bridged by dppm ligands. Solids [1]Cl_2_·8.5H_2_O and [1](OTf)_2_·H_2_O exhibited brilliant green and yellow-green emissions, which were attributed to phosphorescence. The difference in the emission wavelengths between [1]Cl_2_·8.5H_2_O and [1](OTf)_2_·H_2_O (ca. 30 nm) was likely due to the slight but appreciable difference in their Au···Au distances (3.03 Å for [1]Cl_2_·8.5H_2_O vs. 2.98 Å for [1](OTf)_2_·H_2_O). Remarkably, the phosphorescence for both [1]Cl_2_·8.5H_2_O and [1](OTf)_2_·H_2_O was found to have an excellent quantum yield of more than 95%. This high quantum yield appears to be due to the molecular rigidity around two Au^I^ ions tightly bridged by three dppm ligands, together with the multiple intermolecular interactions in crystal, which completely prevented a nonradiative deactivation. The molecular rigidity was supported by the DFT calculations, which revealed a quite small structural difference between the ground singlet (S_0_) and triplet excited (T) states in the optimized molecular structure in [1]Cl_2_ (Supplementary Fig. 15). More remarkably, [1]Cl_2_·8.5H_2_O showed a two-step thermal transformation from its green-emissive crystalline phase to the blue-emissive crystalline phase ([2]Cl_2_) via the yellow-emissive amorphous phase ([1]Cl_2_), which was induced by the loss of water molecules of crystallization and the subsequent dissociation of a dppm ligand. The reverse conversion from [2]Cl_2_ to [1]Cl_2_·8.5H_2_O by mechanical grinding in the presence of water via [1]Cl_2_ was also recognized. To our knowledge, this is the first phosphorescent system with a high quantum yield of >95% that illustrates a reversible, crystalline-amorphous-crystalline thermal transformation accompanied by drastic emission color changes. Such a thermal transformation was not observed for [1](OTf)_2_·H_2_O, which possesses a rigid crystalline framework, sustained by multiple cation-cation and cation-anion interactions without the mediation of water molecules. Thus, it is reasonable to consider that the water-molecule-mediated crystal structure in [1]Cl_2_·8.5H_2_O, which was converted to the loosely packed amorphous structure in [1]Cl_2_ via the removal of water molecules, together with the formation of the closely packed, non-hydrated crystal structure in [2]Cl_2_ via the removal of a dppm ligand, is responsible for this unique thermal transformation. Finally, the present study demonstrated that trigonal-planar Au^I^ species are highly available for the future design and creation of functional luminescent materials.

## Methods

### Materials and equipment

All reagents and solvents used in synthetic studies were commercially available and used as supplied without further purification.

The IR spectra were recorded on a JASCO FT/IR-4100 infrared spectrophotometer using KBr disks at room temperature. The elemental analysis (C, H) was performed at Osaka University using YANACO CHN coda MT-5 or MT-6. The ^1^H and ^31^P NMR spectra in solution were measured using a JEOL EX-500 spectrometer at the probe temperature, using tetramethylsilane (TMS, δ 0.0 ppm) as the internal standard for ^1^H NMR measurements and triphenyl phosphate (δ −17.6 ppm) as the external standard for ^31^P NMR measurements. The ^1^H and ^31^P NMR spectra are illustrated in Supplementary Figs 16 and 17, respectively. The magic angle spinning (MAS) ^31^P NMR spectra in the solid state were measured using a Chemagnetics CMX300 spectrometer at the probe temperature and spun at 7 kHz for [1]Cl_2_∙8.5H_2_O and at 6 kHz for [1]Cl_2_ and [2]Cl_2_, using triphenyl phosphate as the external standard. The TG and DTA measurements were measured using a SHIMADZU DTG-60 analyzer. The PXRD patterns were recorded using a BRUKER D2 PHASER at room temperature. High-quality PXRD patterns for structural determination were recorded at room temperature in transmission mode using a diffractometer equipped with a blue imaging plate detector at the SPring-8 BL19B2 beamline. The crystals were placed in 0.3 mm glass capillary tubes. The samples were rotated during the measurements. The diffraction patterns were collected using a large Debye−Scherrer camera. The powder simulation patterns were generated from the single-crystal X-ray structures using Mercury 3.0.

*Preparation of* [*Au*_*2*_(*dppm*)_*3*_]*Cl*_*2*_*·8.5H*_*2*_*O* ([1]*Cl*_*2*_*·8.5H*_*2*_*O*). To a white suspension containing 50 mg (0.059 mmol) of [Au_2_Cl_2_(dppm)] in MeOH (5 mL) was added a solid sample of dppm (46 mg, 0.12 mmol). The mixture was sonicated for 1 min, producing a clear yellow solution. H_2_O (2 mL) was added to the yellow solution, which was allowed to stand at room temperature for 5 days. The resulting pale yellow block crystals suitable for X-ray analysis were collected via filtration. Yield: 88 mg (85%). Anal. Found: C, 50.88; H, 4.65%. Calcd for [Au_2_(dppm)_3_]Cl_2_∙8.5H_2_O = C_75_H_83_O_8.5_P_6_Au_2_Cl_2_: C, 50.86; H, 4.72%. IR spectrum (cm^−1^, KBr disk): 1436 (ν_Ph_), 1097 and 784, 694 (ν_P–Ph_). ^1^H NMR spectrum (ppm from TMS, CD_3_OD): 7.84–7.79 (m, 8H), 7.59–7.50 (m, 12H), 3.98 (dd, 2H, *J*_1_ = 10.1 Hz, *J*_2_ = 4.6 Hz), 2.92–2.85 (m, 6H), 2.66 (dd, 2H, *J*_1_ = 16.7 Hz, *J*_2_ = 4.8 Hz). ^31^P NMR spectrum (ppm from 80% H_3_PO_4_, CD_3_OD): 37.085.

*Preparation of* [*Au*_*2*_(*dppm*)_*3*_](*OTf* )_*2*_*·H*_*2*_*O* ([1](*OTf* )_*2*_·*H*_*2*_*O*). To a white suspension containing 50 mg (0.059 mmol) of [Au_2_Cl_2_(dppm)] in MeOH (5 mL) was added a solid sample of dppm (46 mg, 0.12 mmol). The mixture was sonicated for 1 min, producing a clear yellow solution. A solution containing 25 mg (0.145 mmol) of NaOTf in a mixture of H_2_O (1 mL) and MeOH (7 mL) was added to the yellow solution, followed by allowing it to stand at room temperature for 2 days. The resulting pale-yellow plate crystals suitable for X-ray analysis were collected via filtration. Yield: 97 mg (88%). Anal. Found: C, 49.86; H, 3.69%. Calcd for [Au_2_(dppm)_3_](OTf)_2_∙H_2_O = C_77_H_68_O_7_S_2_P_6_Au_2_F_6_: C, 49.64; H, 3.68%. IR spectrum (cm^−1^, KBr disk): 1436 (ν_Ph_), 1097 and 775, 693 (ν_P−Ph_).

*Thermal conversion of* [1]*Cl*_*2*_*∙8.5H*_*2*_*O*. A pale yellow crystalline powder of [1]Cl_2_∙8.5H_2_O was heated at 373 K for 30 min under vacuum to produce a dehydrated sample of [1]Cl_2_. During heating, the color of the powder changed from pale-yellow to yellow. The dehydrated sample of [1]Cl_2_ was further heated at 399 K for 30 min. During the second heating, the color of the powder changed from yellow to white. The samples thus obtained were used for the emission and powder X-ray diffraction measurements at ambient temperature.

### Luminescence measurements

The luminescence spectra were recorded by a JASCO FP-8500 spectrometer at room temperature in the solid state or in solution, using a Xe lamp as the light source. The internal emission quantum yields (Φ) were obtained via the absolute measuring method using an integrating sphere unit (JASCO ILFC-847), the internal surface of which was coated with highly reflective Spectralon. The ESC-842 Calibrated light source (WI) and the ESC-843 Calibrated light source (D2) were used to calibrate the emission intensities to measure the absolute quantum yields. The accuracy of this instrument was confirmed using a Rhodamine B ethylene glycol solution. The measurement was performed according to the following protocol. First, nothing was set on the sample cell holder in the integrating sphere, and then, the spectrum of the incident light was measured. The observed peak area was defined as the area from incident light, A_0_ (equivalent number of photons in the incident light). Second, the sample on the sample holder was set in the integrating sphere, and the emission spectra of the sample were measured. The obtained excitation wavelength peak area was defined as the area scattered from the sample, A_1_ (equivalent number of photons that were not absorbed), and peak area in the emission wavelength range was defined as the area emitted from the sample, A_2_. Finally, the internal emission quantum yields (Φ) were calculated using the following equation: Φ = A_2_/(A_0_ – A_1_). We have measured Φ values for more than 10 samples of [1]Cl_2_·8.5H_2_O or [1](OTf)_2_·H_2_O. As a result, the observed Φ values varied in the range of 99–104% for [1]Cl_2_·8.5H_2_O and 99–101% for [1](OTf)_2_·H_2_O. The fluctuations in the Φ values (maximum of 5%) should be regarded as the random error of measurements. In addition, we have measured the absolute quantum yields of Quinine sulfate in a degassed 0.5 M H_2_SO_4_ using the same instrument and the same sample cell in order to evaluate the accuracy of the absolute quantum yields observed by the instrument. The observed quantum yield values of 55–57% were in good agreement with the reported value of 55%^24^. Considering aforementioned results, we determined that the observed Φ values contain 5% error.

Emission lifetime measurements were recorded using a Hamamatsu Photonics, C4334 system equipped with a streak camera as a photo detector and a nitrogen laser for the 337 nm excitation.

The data of the emission data are summarized in Supplementary Table 1.

### X-ray crystal structure determination

The single crystal X-ray diffraction measurements were performed using a Rigaku FR-E Superbright rotating-anode X-ray source with Mo-target (λ = 0.71075 Å), equipped with a Rigaku RAXIS VII imaging plate as a detector, at 200 K. The intensity data were collected via the *ω*-scan technique and empirically corrected for absorption. The structures of the complexes were solved by direct methods using SHELXS2014^25^. The structure refinements were carried out using full matrix least-squares (SHELXL2014)^25^. The data are summarized in Supplementary Table 2.

For [1]Cl_2_·8.5H_2_O, one third of the [Au_2_(dppm)_3_]^2+^ cation, two thirds of the Cl^−^ anion, and two and five sixths of the H_2_O molecules were crystallographically independent. All dppm ligands in the crystal were disordered at two positions, with an occupancy factor of 0.5. All of the benzene rings of dppm ligands were treated using AFIX 66 constraints and SIMU restraints. Some EADP restraints were applied to model the disordered chlorine and water molecules. All non-hydrogen atoms, except water molecules, were refined anisotropically. Hydrogen atoms were included in the calculated positions, except those of H_2_O molecules. For [1](OTf)_2_·H_2_O, two [Au_2_(dppm)_3_]^2+^ cations, four OTf^−^ anions, and two water molecules were crystallographically independent. All of the benzene rings of dppm ligands were treated using AFIX 66 constraints. All non-hydrogen atoms, except one OTf^−^ anion, one benzene ring, and one water molecule, which were disordered into two parts with occupancy factors of 0.5, were refined anisotropically. Hydrogen atoms were included in the calculated positions. Three of the four OTf^−^ anions were modeled to adopt ideal conformations using FRAG commands. These anions were treated using EADP or DELU restraints.

### *Ab Initio* crystal structure determination of [2]Cl_2_

A high-quality PXRD pattern of [2]Cl_2_ was recorded at 298 K in transmission mode [0.3 mm capillary; synchrotron radiation *λ* = 1.3 Å; 2*θ* range, 0.00 to 78.09°; step size, 0.01°; data collection time, 25 min] using a diffractometer equipped with a blue imaging plate detector at the BL19B2 beam line, SPring-8.

The PXRD pattern of [2]Cl_2_ was indexed using the program DICVOL^26^ to produce a monoclinic unit cell (*a* = 20.588 Å, *b* = 18.129 Å, *c* = 12.988 Å, *β* = 98.892˚, *V* = 4789.27 Å^3^) with good figure of merit. The space group was assigned based on systematic absences as *P2*_*1*_*/n*. Unit cell and profile refinement were carried out using the Pawley method and led to a good fit (*χ*^2^ = 3.87) for these lattice parameters and the space group. The structure solution was obtained using the simulated annealing method with the program DASH^27^. Two rigid groups: [Au_2_(dppm)_2_], in which P-C_phenyl_ were allowed to rotate, and two Cl^−^ molecules, in asymmetric units and *Z* = 4 for space group *P*2_1_/*n*, were introduced using a constrained Z-matrix description. During annealing, 26 runs of 1 × 10^7^ Monte Carlo moves were performed. The best structure obtained (Profile *χ*^2^ = 24.45) was taken as the starting structural model for Rietveld refinement. The Rietveld refinement^28^ of [2]Cl_2_ was performed using the programs RIETAN-FP^29^ and VESTA^30^, introducing disorder for each Cl^−^ anion. Restraints but no constraints for all bond lengths were employed to maintain the molecular geometry. Atomic displacement parameters were refined isotopically. Absorption collection was applied using the RIETAN-FP Program. Final Rietveld refinement result: *a* = 20.5723(6) Å, *b* = 18.1157(5) Å, *c* = 12.975(3) Å, *β* = 98.900(2)°, *V* = 4777.5(2) Å^3^, *R*_wp_ = 8.860% (*R*_e_ = 10.850%), *R*_p_ = 6.403%, *R*_B_ = 6.775%, *R*_F_ = 6.605%; 5,801 profile points (2*θ* range, 2 to 60°); 282 refined variables. The result is shown in Supplementary Fig. 9.

### *Ab Initio* crystal structure determination of [Au_2_Cl_2_(dppm)_2_]

A high-quality PXRD pattern of [Au_2_Cl_2_(dppm)_2_], which was prepared from [Au_2_(dppm)_2_Cl_2_]·(acetone) after being heated at 399 K, was recorded at 298 K in transmission mode [0.3 mm capillary; synchrotron radiation *λ* = 1.3 Å; 2*θ* range, 0.00 to 78.09°; step size, 0.01°; data collection time, 20 min] using a diffractometer equipped with a blue imaging plate detector at the BL19B2 beam line, SPring-8.

The PXRD pattern of [Au_2_Cl_2_(dppm)_2_] was indexed using the program DICVOL^26^ to produce a triclinic unit cell (*a* = 20.481 Å, *b* = 14.008 Å, *c* = 11.237 Å, *α* = 66.65°, *β* = 109.83°, *γ* = 125.98°, *V* = 2370.44 Å^3^) with good a figure of merit. The space group was assigned as *P*-1 based on systematic absences. Unit cell and profile refinement were carried out using the Pawley method and led to a good fit (*χ*^2^ = 3.10) for these lattice parameters and the space group. The structure solution was carried out using the simulated annealing method with the program DASH. Two rigid groups: [Au(dppm)]Cl, in which P-C_phenyl_ were allowed to rotate in an asymmetric unit and *Z* = 2 for space group *P*-1, were introduced using a constrained Z-matrix description. During annealing, 26 runs of 1 × 10^7^ Monte Carlo moves were performed. The best structure obtained (Profile *χ*^2^ = 20.92) was taken as the starting structural model for Rietveld refinement. Absorption collection was applied using the RIETAN-FP Program. The Rietveld refinement of [Au_2_Cl_2_(dppm)_2_] was performed using the programs RIETAN-FP and VESTA. Restraints but no constraints for all bond lengths were employed to maintain the molecular geometry. Atomic displacement parameters were refined isotopically. Final Rietveld refinement result: *a* = 20.4758(0) Å, *b* = 14.0049(0) Å, *c* = 11.2323(0) Å, *α* = 66.63(0)°, *β* = 109.68(0)°, *γ* = 125.96(0)°, *V* = 2368.42(0) Å^3^, *R*_wp_ = 5.150% (*R*_e_ = 10.504%), *R*_p_ = 3.908%, *R*_B_ = 1.505%, *R*_F_ = 0.794%; 5,701 profile points (2*θ* range, 3 to 60°); 242 refined variables. The result is shown in Supplementary Fig. 10.

### DFT calculations

DFT calculations for the [1]Cl_2_·H_2_O system were performed using the Gaussian 09 program^31^ with the PBE0 functional. The def2-tzvppd basis set was applied for Au and P atoms in [1]^2+^. 6–311G(d) basis sets were applied for bridging C atoms between two P atoms in [1]^2+^, and 6–31G and STO-3G basis sets were applied for C and H atoms in phenyl rings in [1]^2+^, respectively. Additionally, 6–311+G* basis sets for Cl^−^ and H_2_O molecules were used. The single-point and time-dependent DFT calculations were carried out for [1]Cl_2_·H_2_O. The initial structural parameters were taken from the single-crystal X-ray structure of [1]Cl_2_·8.5H_2_O. The contour plots of HOMO-12, HOMO-11 and LUMO are shown in Supplementary Fig. 3. The calculated absorption spectrum of [1]Cl_2_·H_2_O is illustrated in Supplementary Fig. 4. The major components in the calculated absorption spectrum were summarized in Supplementary Table 3. The optimized molecular structures in the singlet ground state and triplet excitation state are demonstrated in Supplementary Fig. 15.

## Additional Information

**Accession codes**: The X-ray crystallographic coordinates for the structure reported in this Article have been deposited at the Cambridge Crystallographic Data Centre (CCDC) under deposition number CCDC 1437780-1437781 (powder data), 1437852-1437853 (single-crystal data). These data can be obtained free of charge from The Cambridge Crystallographic Data Centre via www.ccdc.cam.ac.uk/data_request/cif. 

**How to cite this article**: Igawa, K. *et al*. Crystalline-Amorphous-Crystalline Transformation in a Highly Brilliant Luminescent System with Trigonal-Planar Gold(I) Centers. *Sci. Rep*. **6**, 26002; doi: 10.1038/srep26002 (2016).

## Supplementary Material

Supplementary Information

## Figures and Tables

**Figure 1:**
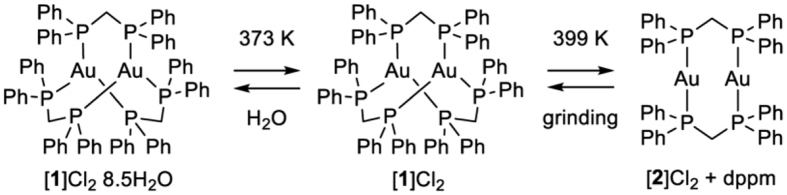
Reversible two-step conversion of [1]Cl_2_·8.5H_2_O.

## References

[b1] Keefe M. H., Benkstein K. D. & Hupp J. T. Luminescent sensor molecules based on coordinated metals: a review of recent developments. Coord. Chem. Rev. 205, 201–228 (2000).

[b2] Zhang X. . Recent advances in mechanochromic luminescent metal complexes. J. Mater. Chem. C 1, 3376–3390 (2013).

[b3] Wenger O. S. Vapochromism in Organometallic and Coordination Complexes: Chemical Sensors for Volatile Organic Compounds Chem. Rev. 113, 3686–3733 (2013). 23317416 10.1021/cr300396p

[b4] Katz M. J., Ramnial T., Yu H. & Leznoff D. B. Polymorphism of Zn[Au(CN)_2_]_2_ and Its Luminescent Sensory Response to NH_3_ Vapor. J. Am. Chem. Soc. 130, 10662–10673 (2008). 18642808 10.1021/ja801773p

[b5] Jobbágy C. . stimuli-responsive double-stranded digold(I) helicate. CrystEngComm 16, 3192–3202 (2014).

[b6] Saitoh M., Balch A. L., Yuasa J. & Kawai T. Effects of Counter Anions on Intense Photoluminescence of 1-D Chain Gold(I) Complexes. Inorg. Chem. 49, 7129–7134 (2010). 20583819 10.1021/ic100948m

[b7] Hashimoto Y. . Synthesis, Structures, and Luminescence Properties of Interconvertible Au^I^_2_Zn^II^ and Au^I^_3_Zn^II^ Complexes with Mixed Bis(diphenylphosphino)methane and d-Penicillaminate. Inorg. Chem. 52, 14368–14375 (2013). 24256143 10.1021/ic4024629

[b8] Liang J. . Aggregation-induced emission (AIE) behavior and thermochromic luminescence properties of a new gold(I) complex. Chem. Commun. 49, 3567–3569 (2013). 10.1039/c3cc00157a 23527379

[b9] Ito H. . Mechanical stimulation and solid seeding trigger single-crystal-to-single-crystal molecular domino transformations. Nat. Commun. 4, 2009–2014 (2013). 23764759 10.1038/ncomms3009

[b10] Pyykkö P. Strong Closed-Shell Interactions in Inorganic Chemistry. Chem. Rev. 97, 597–636 (1997). 11848883 10.1021/cr940396v

[b11] Schmidbaur H. & Schier A. A briefing on aurophilicity. Chem. Soc. Rev. 37, 1931–1951 (2008). 18762840 10.1039/b708845k

[b12] King C., Wang J., Khan M. N. I. & Fackler J. P. Jr. Luminescence and Metal-Metal Interactions in Binuclear Gold(I) Compounds. Inorg. Chem. 28, 2145–2149 (1989).

[b13] Tanase T. . Strongly Luminous Tetranuclear Gold(I) Complexes Supported by Tetraphosphine Ligands, *meso*- or *rac*-Bis[(diphenylphosphinomethyl)phenylphosphino]methane. Chem. Eur. J. 20, 1577–1596 (2014). 24403217 10.1002/chem.201303729

[b14] Li D., Che C., Kwong H. & Yam V. W. W. Photoinduced C-C Bond Formation from Alkyl Halides catalysed by Luminescent Dinuclear Gold(I) and Copper(I) Complexes. J. Chem. Soc., Dalton trans. 3325–3329 (1992).

[b15] Shieh S. Li D., Peng S. & Che C. Synthesis, Photophysical Properties and Crystal Structure of a Luminescent Binuclear Three-coordinated Gold(I) Complex without Metal-Metal Interaction. J. Chem. Soc., Dalton trans. 195–196 (1993).

[b16] Yam V. W. & Lee W. Synthesis, Spectroscopy and Excited-state Redox Properties of Novel Luminescent Trinuclear Three-co-ordinate Gold(I) Phosphine Complexes. J. Chem. Soc., Dalton trans. 2097–2100 (1993).

[b17] Wei Z. . Rigidifying Fluorescent Linkers by Metal–Organic Framework Formation for Fluorescence Blue Shift and Quantum Yield Enhancement. J. Am. Chem. Soc. 136, 8269–8276 (2014). 24819882 10.1021/ja5006866

[b18] Al-Baker S., Hill W. E. & McAuliffe C. A. Novel Ring Compounds of Bidentate Phosphines with Gold(I). Two-, Three-, and Four-co-ordination. J. Chem. Soc. Dalton trans. 2655–2659 (1985).

[b19] Mirabelli C. K. . Antitumor Activity of Bis(diphenylphosphino)alkanes, Their Gold(I) Coordination Complexes, and Related Compounds. J. Med. Chem. 30, 2181–2190 (1987). 3681888 10.1021/jm00395a004

[b20] Nakamoto K. Infrared and Raman Spectra of Inorganic and Coordination Compounds, part A, 5^th^ ed., Sec. II, 153–320 (Wiley, 2008).

[b21] Hong M. . Syntheses, structures and spectroscopic properties of [Ag(dppm)(O_2_CCH_2_Ph)]_2_ and [Ag_2_(dppm)_3_](NO_3_)_2_·3H_2_O. Polrhedron 16, 11, 1957–1962 (1977).

[b22] Gimeno M. C. & Laguna A. Three- and Four-Coordinate Gold(I) Complexes. Chem. Rev. 97, 3, 511–522 (1997). 11848881 10.1021/cr960361q

[b23] Schmidbaur H.. & Gold-Komplexe von Diphosphinomethanen I. I. Synthese und Kristallstruktur achtgliedriger Ringverbindungen von Gold(I) mit Au–Au-Wechselwirkung. Chem. Ber. 110, 2751–2757 (1977).

[b24] Birks J. B. Fluorescence quantum yield measurement. J. Res. Natl. Bur. Stand. A 80, 389–399 (1976). 10.6028/jres.080A.038 PMC5293345 32196267

[b25] Sheldrick G. M. A short history of SHELX. Acta Cryst. A 64, 112–122 (2008). 10.1107/S0108767307043930 18156677

[b26] Boultif A. & Louër D. Indexing of powder diffraction patterns for low-symmetry lattices by the successive dichotomy method. J. Appl. Crystallogr. 24, 987–993 (1991).

[b27] David W. I. F. . DASH: a program for crystal structure determination from powder diffraction data. J. Appl. Crystallogr. 39, 910–915 (2006).

[b28] Rietveld H. M. A profile refinement method for nuclear and magnetic structures. J. Appl. Crystallogr. 2, 65–71 (1969).

[b29] Izumi F. & Momma K. Three-dimensional visualization in powder diffraction. Solid State Phenom. 130, 15–20 (2007).

[b30] Momma K. & Izumi F. VESTA: a three-dimensional visualization system for electronic and structural analysis. J. Appl. Crystallogr. 41, 653–658 (2008).

[b31] Frisch M. J. . Gaussian 09, Revision C.01 (2009), Gaussian, Inc., Wallingford CT. URL http://www.gaussian.com/.

