# Supplementary material for: Crystalline-Amorphous-Crystalline Transformation in a Highly Brilliant Luminescent System with Trigonal-Planar Gold(I) Centers
Source: Sci Rep. 2016 May 17;6:26002. doi: 10.1038/srep26002 (PMC4868988; doi:10.1038/srep26002)
Supplement: Supplementary Information [file srep26002-s1.pdf]

Supplementary Information

Crystalline-Amorphous-Crystalline Transformation in a Highly Brilliant Luminescent System with Trigonal-Planar Gold(I) Centers

Kosuke Igawa[1], Nobuto Yoshinari[1], Mitsutaka Okumura[1], Hiroyoshi Ohtsu[2], Masaki Kawano[2,3] & Takumi Konno[1]

[1] Department of Chemistry, Graduate School of Science, Osaka University, Toyonaka, Osaka 560-0043, Japan
[2] The Division of Advanced Materials Science, Pohang University of Science and Technology (POSTECH), San 31, Hyoja-dong, Pohang 790-784, Korea.
[3] Department of Chemistry, Graduate School of Science and Engineering, Tokyo Institute of Technology, 2-12-1 Ookayama, Meguro-ku, Tokyo 152-8550, Japan.

Supplementary Table 1 | Emission (em) data in the solid state.

compounds	em: λ_{max}/ nm [a]	Φ [b]	τ / µs [c]
[**1**]Cl_2·8.5H_2O [d]	513	>0.95	4.51
[**1**]Cl_2·8.5H_2O [e]	523	>0.95	5.71
[**1**]Cl_2 [d]	590	0.52	*f*
[**2**]Cl_2 [d]	473	0.55	*f*
[**1**]$(OTf)_2$·H_2O [d]	540	>0.95	4.15
[**1**]$(OTf)_2$·H_2O [e]	555	>0.95	5.02
[Au_2Cl_2(dppm)$_2$] [d,g]	480	0.69	*f*

a The excitation wavelength was set to 390 nm. *b* Error ± 5%. *c* Determined with excitation at 337 nm. *d* Measured at ambient temperature. *e* Measured at 77 K. *f* Not measured. *g* Heated sample of [Au_2Cl_2(dppm)$_2$]·(acetone).

Supplementary Table 2 | Crystallographic data of [1]Cl_2·8.5H_2O and [1]$(OTf)_2$·H_2O.

	**[1]Cl_2·8.5H_2O**	**[1]$(OTf)_2$·H_2O**
Formula	$C_{75}H_{66}Au_2Cl_2O_{8.5}P_6$	$C_{154}H_{132}Au_4F_{12}O_{14}P_{12}S_4$
Color, form	Pale yellow, block	Pale yellow, plate
Mw	1753.93	3722.33
Crystal system	Cubic	Monoclinic
Space group	*Pa*-3	$P2_1/n$
a/ Å	24.7728(9)	23.6242(4)
b/ Å	24.7728(9)	26.5462(5)
c/ Å	24.7728(9)	23.9188(4)
α (°)	90	90
β (°)	90	101.304(7)
γ (°)	90	90
V/ Å^3	15202.9(10)	14709.3(4)
Z	8	4
T/ K	200(2)	200(2)
F(000)	6928	7344
ρ calcd/ g· cm^{-3}	1.533	1.878
μ(Mo Kα)/ mm^{-1}	4.104	4.242
Crystal size /mm^3	0. 20×0.20×0.20	0.10×0.05×0.05
Limiting indices	$-29 \le h \le 32$,	$-30 \le h \le 30$,
	$-31 \le k \le 32$,	$-34 \le k \le 34$,
	$-29 \le l \le 32$	$-29 \le l \le 30$
R1 (I>2σ(I))[a)]	0.1282	0.0817
wR2 (all data) [b)]	0.2697	0.1666
GOF	1.332	1.071

a) $R1 = \Sigma||F_o|-|F_c|| / \Sigma|F_o|$.

b) $wR2 = [\Sigma(w(F_o^2-F_c^2)^2) / \Sigma w(F_o^2)^2]^{1/2}$.

Supplementary Table 3 | Major components in the calculated absorption spectrum of Au complex.

System	Absorption energy, nm	Excitation Nature [a]
$[Au_2(dppm)_3]Cl_2$	325.2	HOMO-11→LUMO (0.63822)
		HOMO-12→LUMO(-0.15071)
	324.4	HOMO-11→LUMO(0.14028)
		HOMO-12→LUMO(0.64768)

[a] Major coefficients in the CI expansion are in parenthesis.

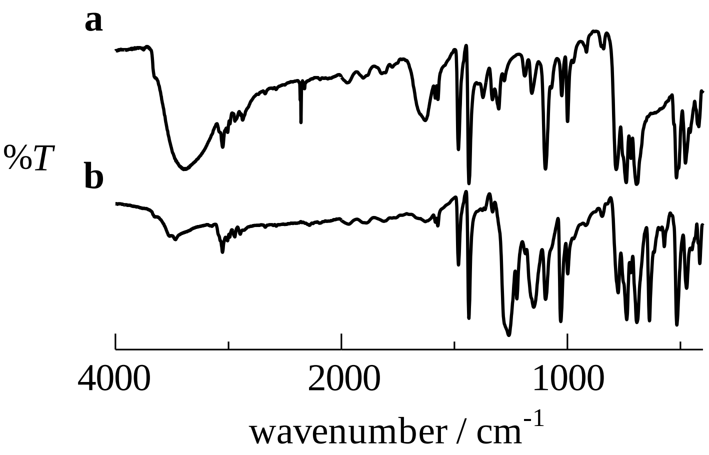

Supplementary Figure 1 | IR spectra of a, [1]Cl_2·8.5H_2O and b, [1]$(OTf)_2$·H_2O.

a

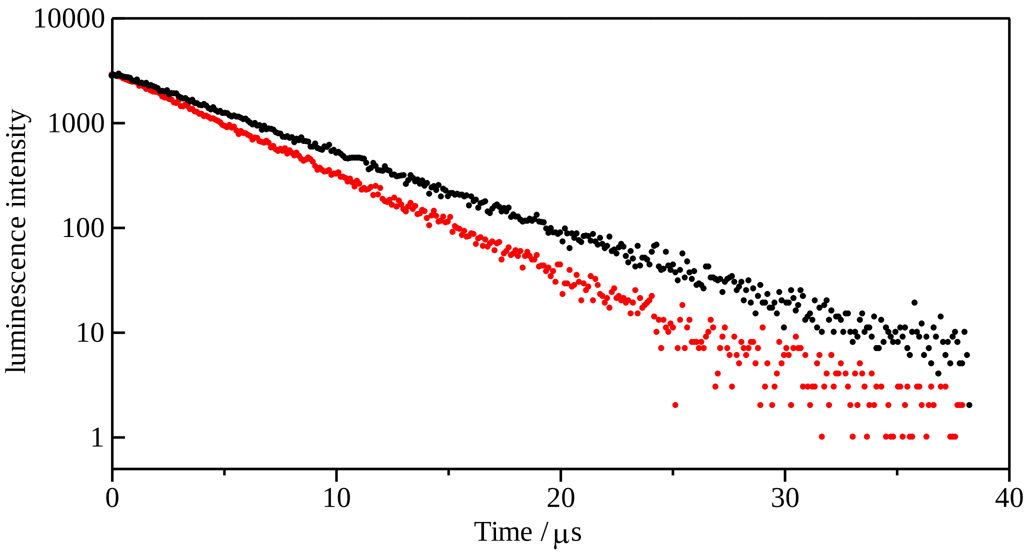

b

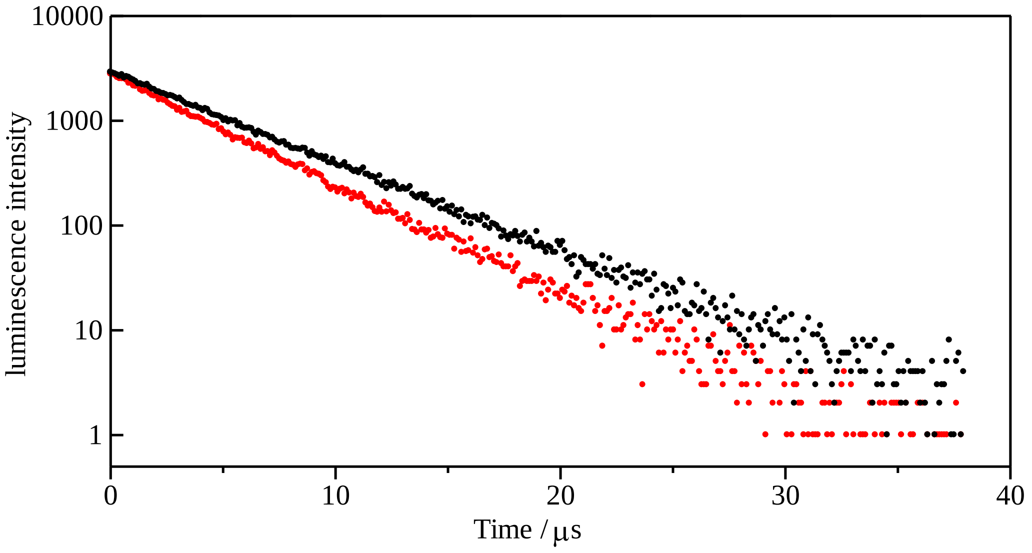

Supplementary Figure 2 | Emission decay of a, [1]$Cl_2 \cdot 8.5H_2O$ (λ_{ex} = 337 nm) and b, [1]$(OTf)_2 \cdot H_2O$ (λ_{ex} = 337 nm). Red and black dots indicate the data measured at room temperature and 77 K.

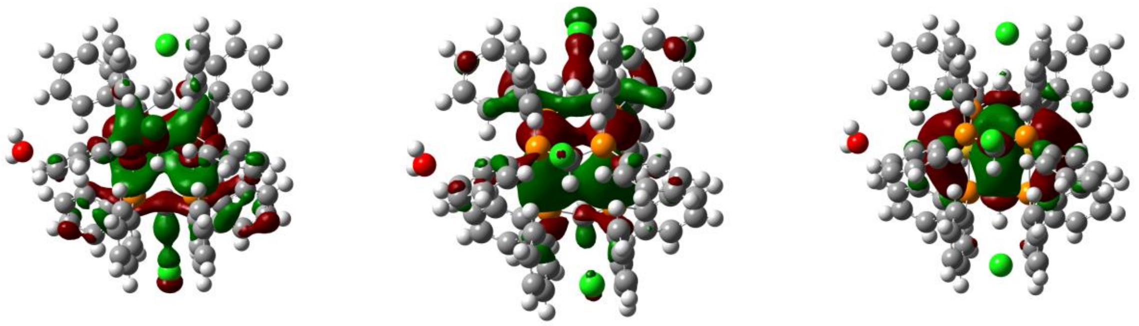

HOMO-12 HOMO-11 LUMO

Supplementary Figure 3 | Contour plots of [1]$Cl_2 \cdot H_2O$.

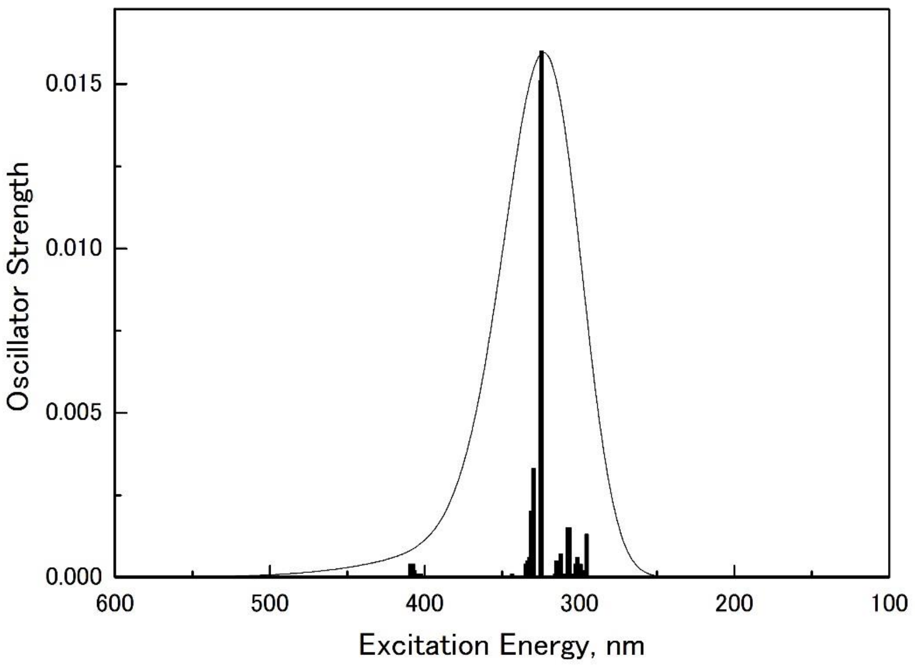

Supplementary Figure 4 | Simulated absorption spectrum of Au complex with 3Cl^- calculated by TD-DFT calculation. The two dominant components in the absorption spectrum were transitions from HOMO-12 to LUMO and from HOMO-11 to LUMO.

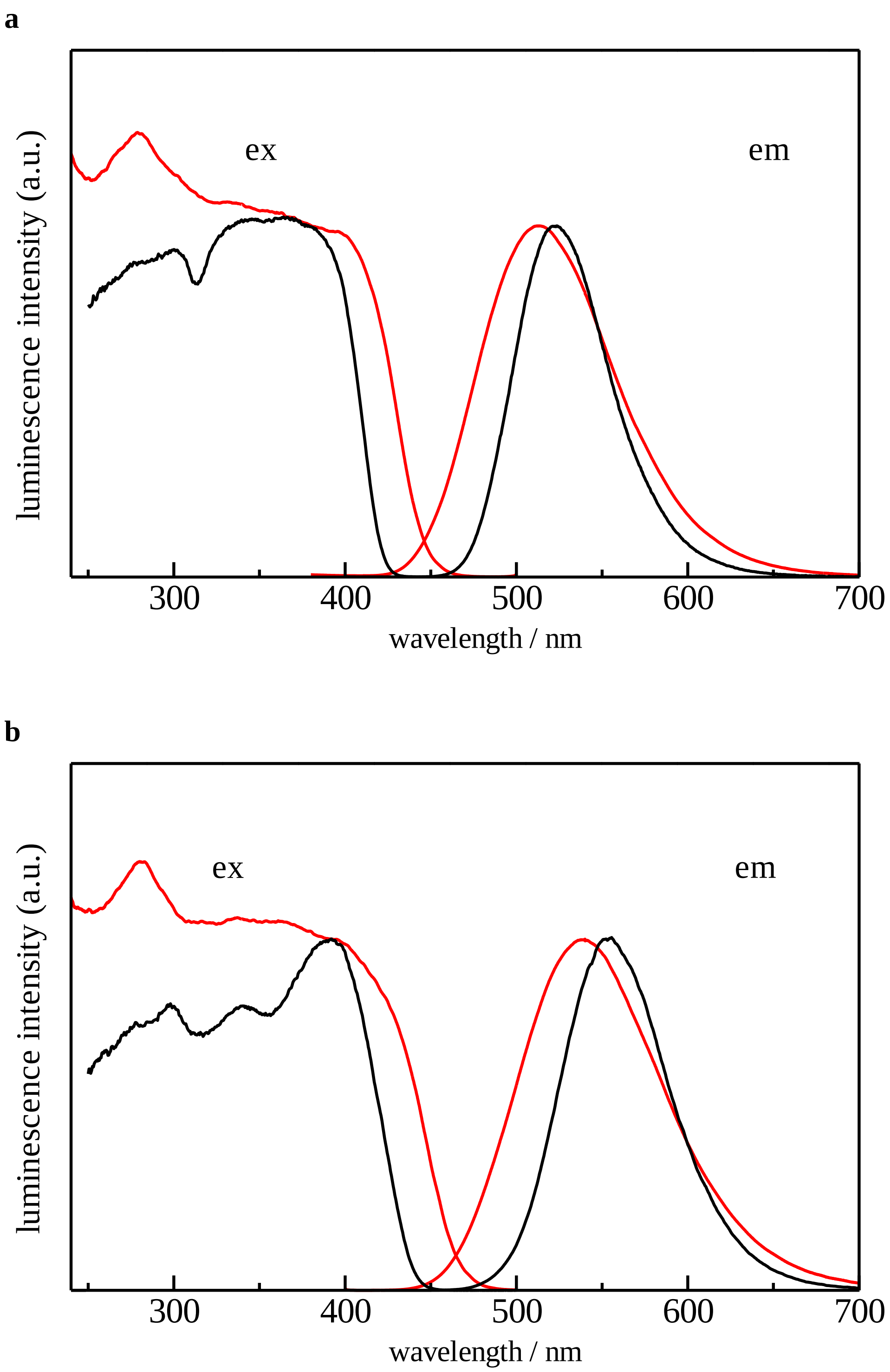

Supplementary Figure 5 | Emission (em) and excitation (ex) spectra of a, [1]Cl_2·8.5H_2O and b, [1]$(OTf)_2$·H_2O. Red and black lines indicate the data measured at room temperature and 77 K. λ_{ex} = 390 nm for all measurements. λ_{em} = 510 nm (room temperature) or 523 nm (77 K) for [**1**]Cl_2·8.5H_2O. λ_{em} = 540 nm (room temperature) or 555 nm (77 K) for [**1**]$(OTf)_2$·H_2O.

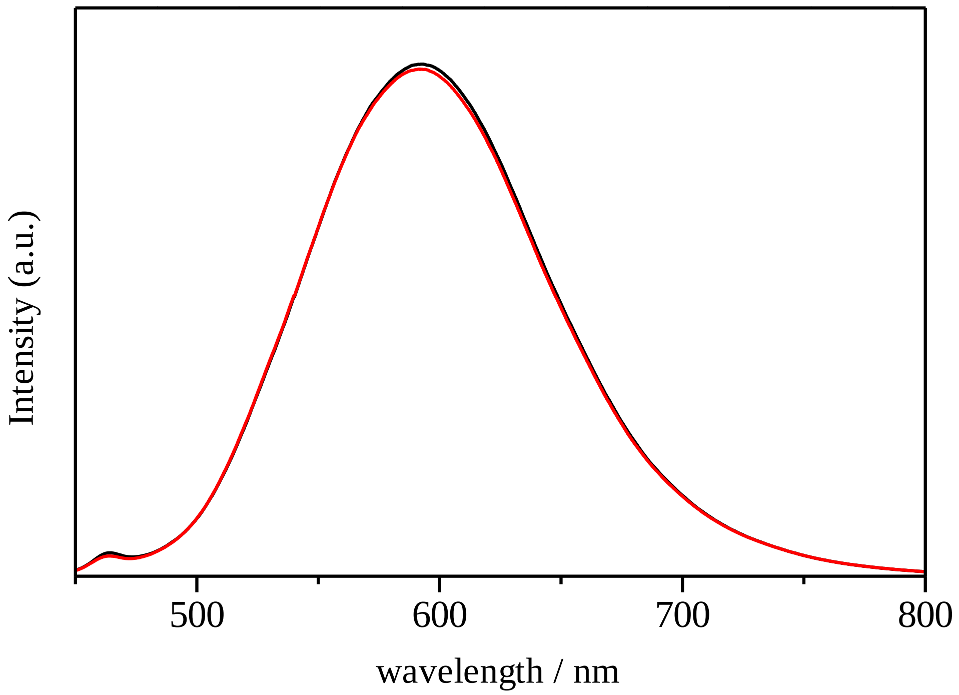

Supplementary Figure 6 | Emission spectra of [1]Cl_2·8.5H_2O (black line) and [1]$(OTf)_2$·H_2O (red line) in MeOH at room temperature (λ_{ex} = 407 nm).

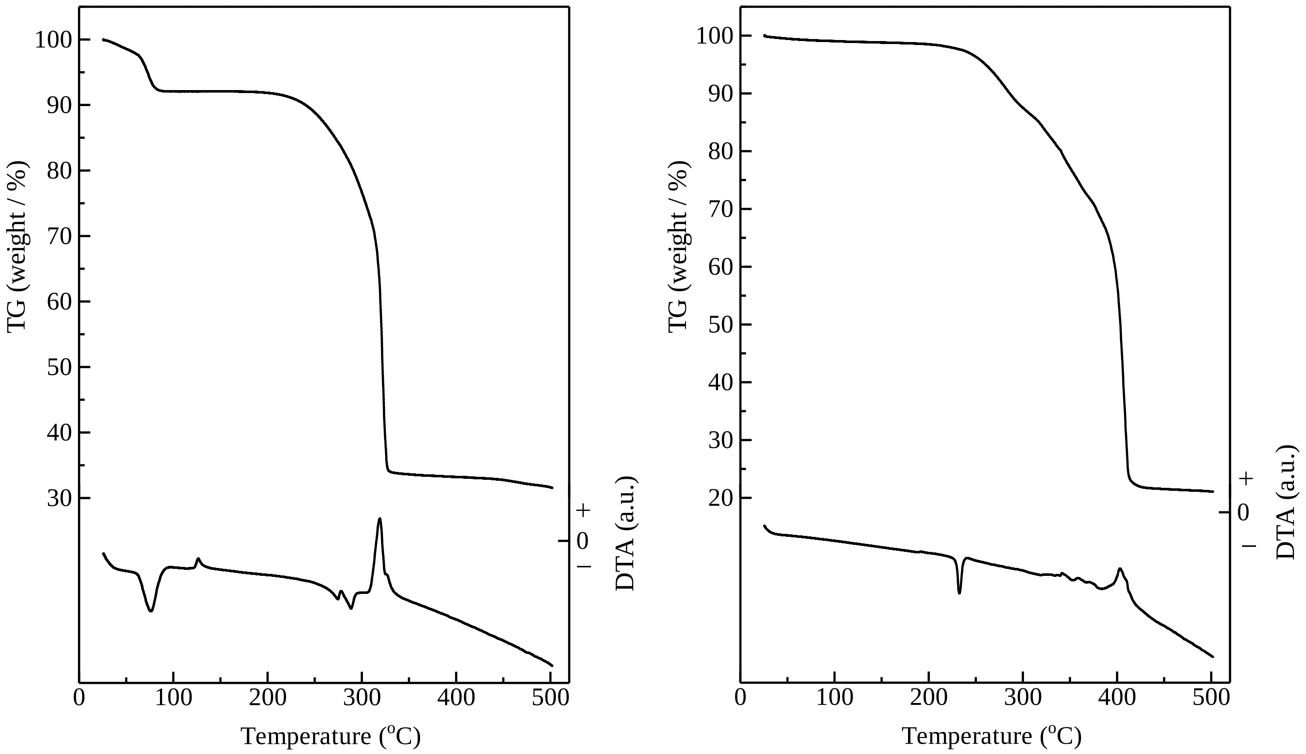

Supplementary Figure 7 | Thermogravimetric (TG) and differential thermal analysis (DTA) curves of [1]Cl_2·8.5H_2O (left) and [1]$(OTf)_2$·H_2O (right).

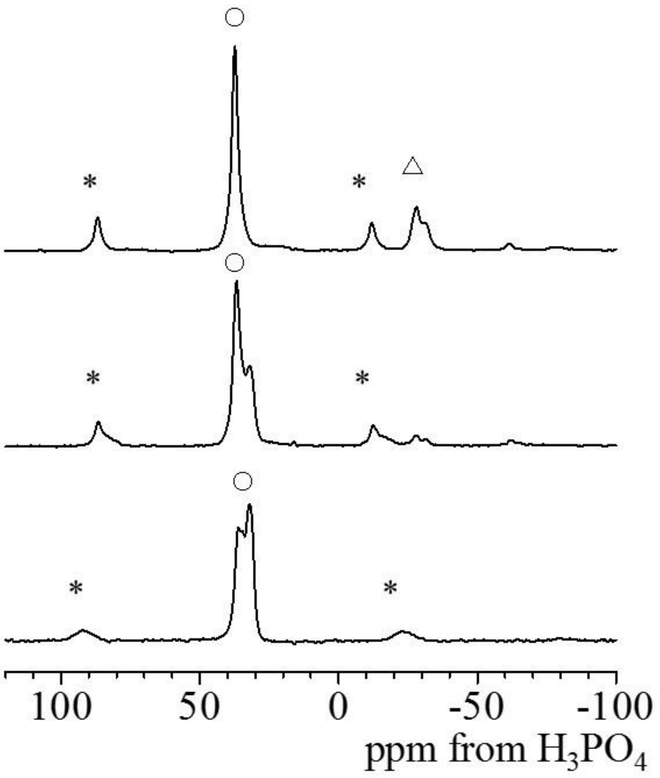

Supplementary Figure 8 | Solid-state MAS ^{31}P spectra of $[1]Cl_2{\cdot}8.5H_2O$, measured at room temperature. Bottom: fresh, middle: heated at 373 K, top: heated at 399 K. Symbols *, °, and Δ indicate side bands, bands due to coordinated dppm, and a band due to free dppm, respectively.

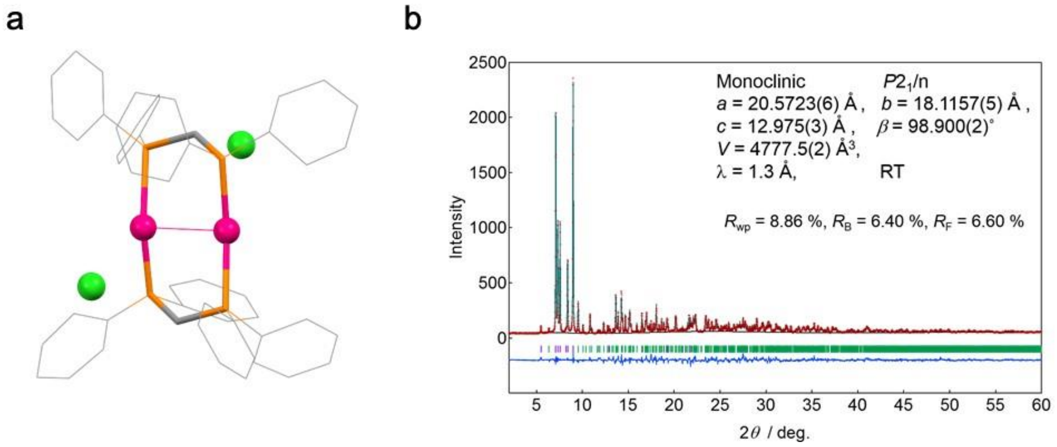

Supplementary Figure 9 | Perspective view of a, [2]Cl_2, which was determined by b, PXRD studies; experimental (red), calculated (black), and difference (blue) PXRD profiles and Bragg positions (green).

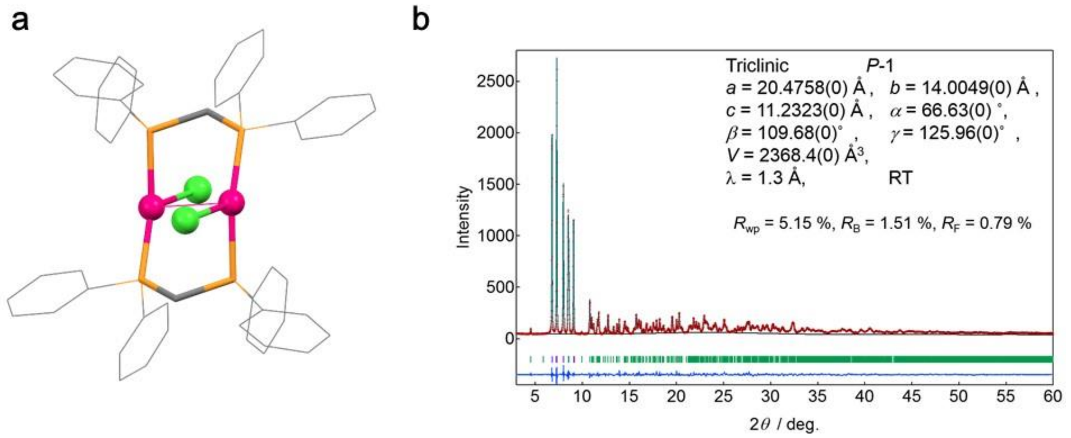

Supplementary Figure 10 | A perspective view of a, [Au_2(dppm)$_2Cl_2$], which was determined by b, PXRD studies; experimental (red), calculated (black), and difference (blue) PXRD profiles and Bragg positions (green). We determine the crystal structure of the heated sample of [Au_2(dppm)$_2Cl_2$]·(acetone). The Rietveld analysis of the powder X-ray diffraction pattern showed that the heated sample is [Au_2(dppm)$_2Cl_2$], where Au centers take a T-shaped structure bound by Cl^- (av. Au–Cl = 3.00 Å).

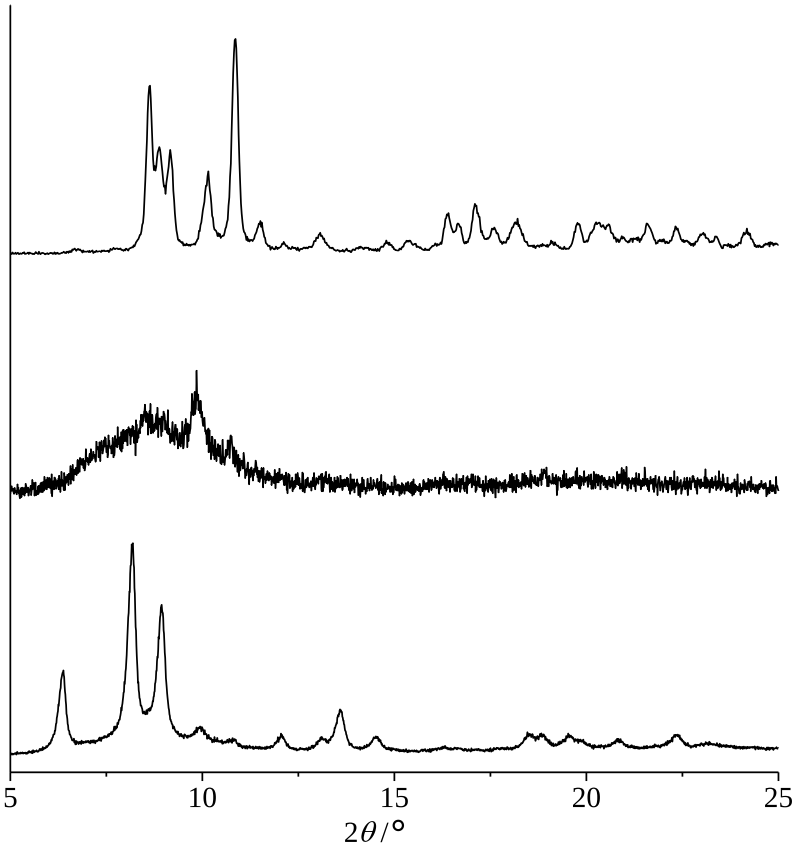

Supplementary Figure 11 | Powder X-ray diffraction in the solid state. Bottom: [**2**]Cl_2 after grinding in water, middle: ground sample of [**2**]Cl_2, top: [**2**]Cl_2.

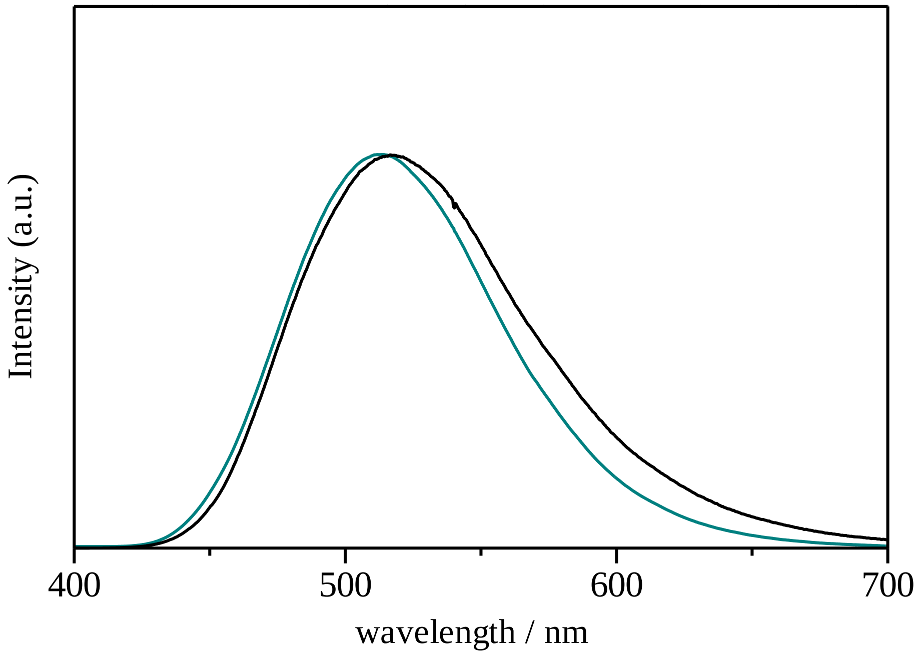

Supplementary Figure 12 | Emission spectra in the solid state. Green line: [**1**]$Cl_2 \cdot 8.5H_2O$, black line: [**2**]Cl_2 after grinding in water. The emission quantum yield of the recovered sample is 85%. The lower quantum yield is likely due to the imperfect restoration of crystallinity resulting from this manual operation.

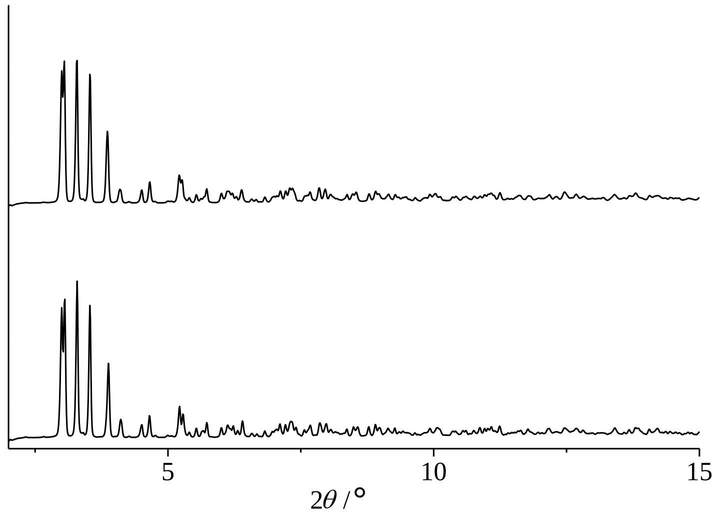

Supplementary Figure 13 | Powder X-ray diffraction of [1]$(OTf)_2 \cdot H_2O$ in the solid state. Bottom: fresh crystals, top: after being heated at 473 K.

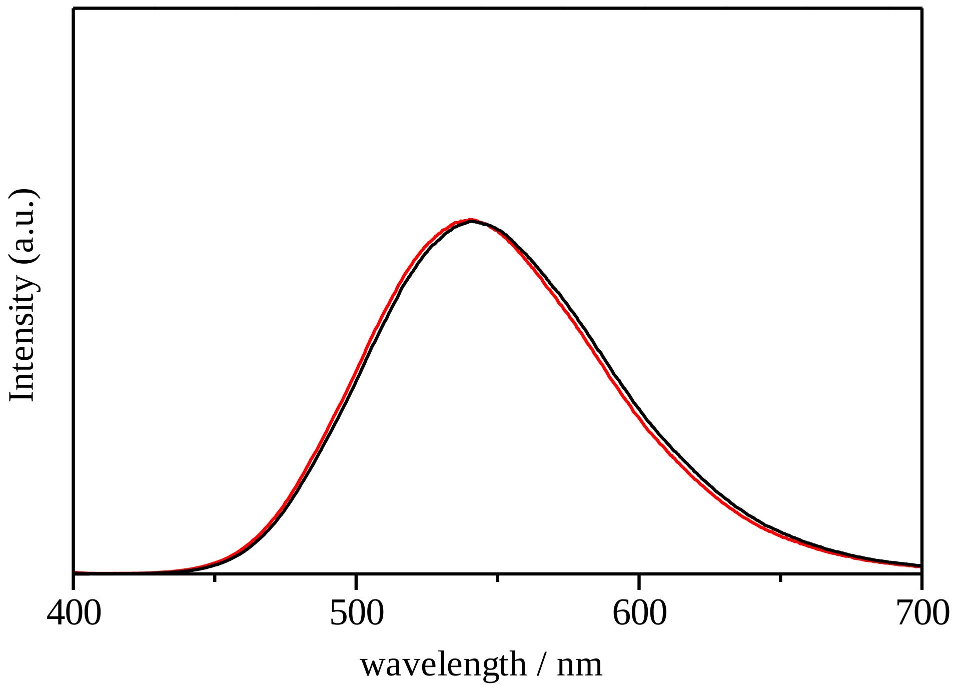

Supplementary Figure 14 | Emission spectra of [1]$(OTf)_2 \cdot H_2O$ in the solid state. Fresh sample (black line) and after being heated at 473 K (red line).

a **b**

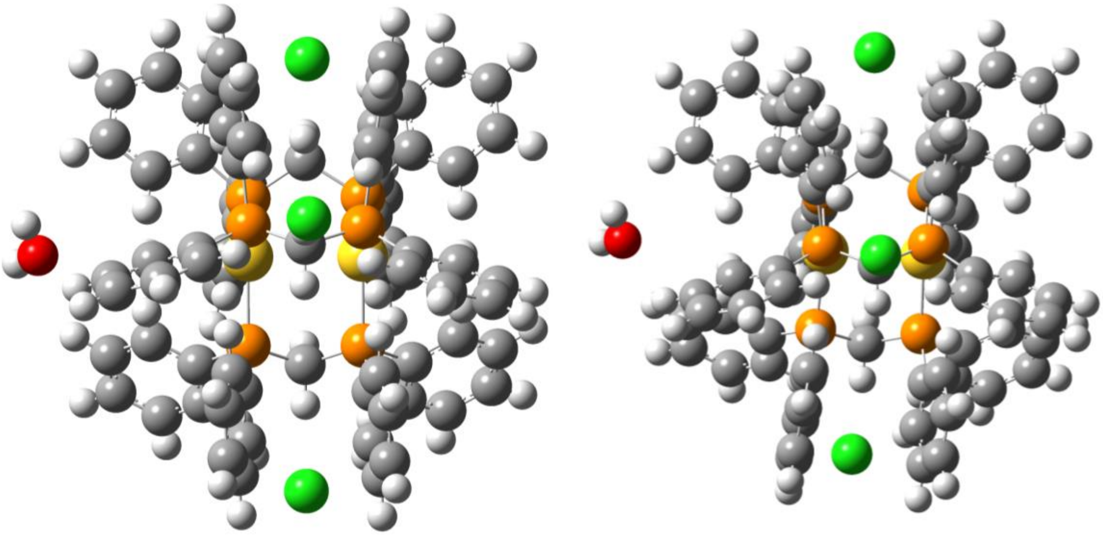

R(Au-Au)=3.073 Å R(Au-Au)=2.877 Å

Supplementary Figure 15 | Optimized structures of a, singlet ground state and b, triplet excitation state of [1]$Cl_2 \cdot H_2O$.

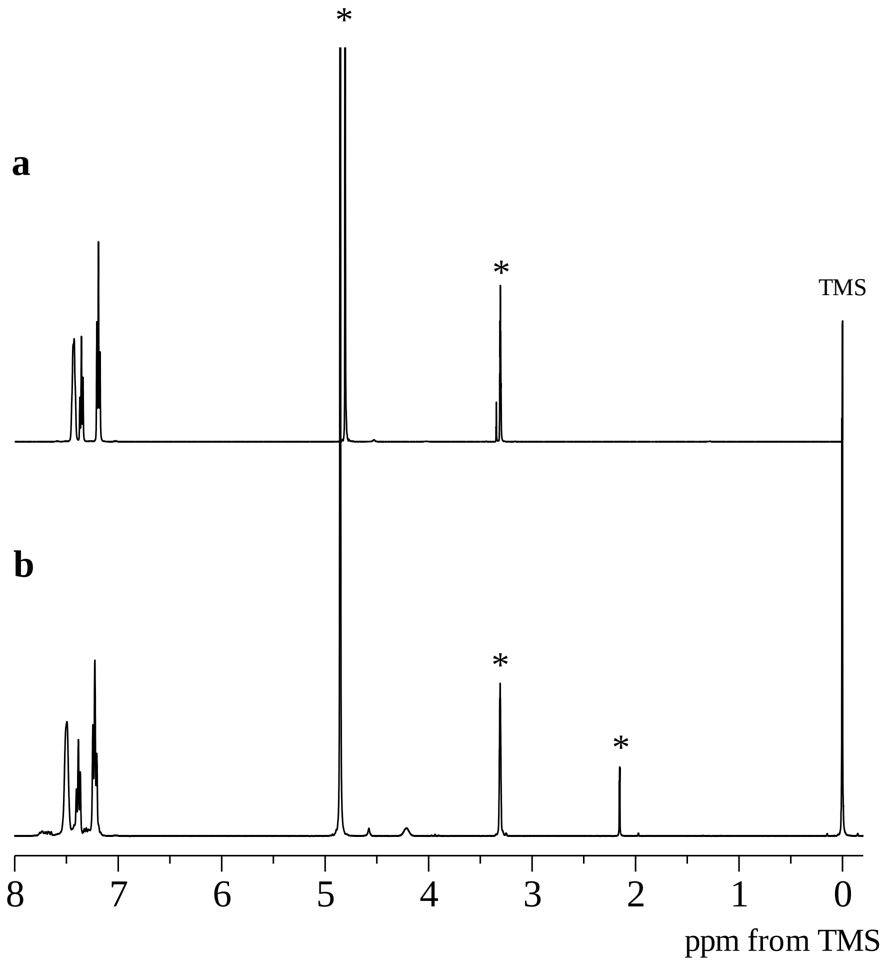

Supplementary Figure 16 | ^{1}H NMR spectrum of a, [1]Cl_2·8.5H_2O and b, [2]Cl_2 in CD_3OD. The symbol (*) indicates solvents.

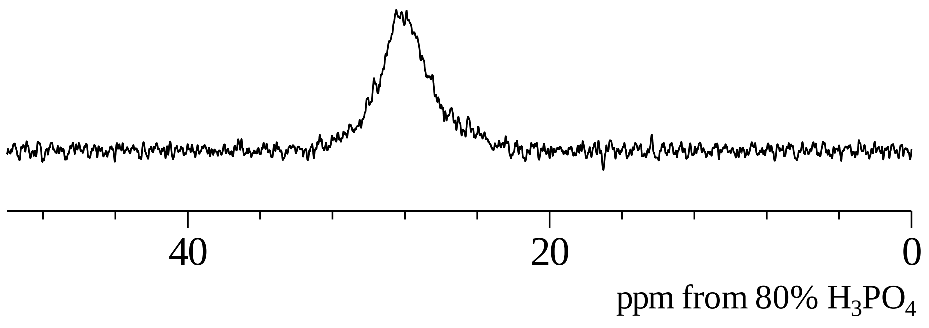

Supplementary Figure 17 | ^{31}P NMR spectrum of [1]Cl_2·8.5H_2O in CD_3OD.
